# Antioxidant mechanisms of mesenchymal stem cells and their therapeutic potential in vitiligo

**DOI:** 10.3389/fcell.2023.1293101

**Published:** 2023-12-21

**Authors:** Rui-lin Yang, Si-yu Chen, Sheng-ping Fu, De-zhi Zhao, Wei-hong Wan, Kang Yang, Wei Lei, Ying Yang, Qian Zhang, Tao Zhang

**Affiliations:** ^1^ Key Laboratory of Cell Engineering of Guizhou Province, Affiliated Hospital of Zunyi Medical University, Zunyi, China; ^2^ Department of Dermatology, Affiliated Hospital of Zunyi Medical University, Zunyi, China; ^3^ Department of Human Anatomy, Zunyi Medical University, Zunyi, China

**Keywords:** vitiligo, oxidative stress, melanocyte, mesenchymal stem cells, reactive oxygen species, antioxidant

## Abstract

Vitiligo is a skin pigmentation disorder caused by melanocyte damage or abnormal function. Reactive oxygen species (ROS) can cause oxidative stress damage to melanocytes, which in turn induces vitiligo. Traditional treatments such as phototherapy, drugs, and other methods of treatment are long and result in frequent recurrences. Currently, mesenchymal stem cells (MSCs) are widely used in the research of various disease treatments due to their excellent paracrine effects, making them a promising immunoregulatory and tissue repair strategy. Furthermore, an increasing body of evidence suggests that utilizing the paracrine functions of MSCs can downregulate oxidative stress in the testes, liver, kidneys, and other affected organs in animal models of certain diseases. Additionally, MSCs can help create a microenvironment that promotes tissue repair and regeneration in areas with oxidative stress damage, improving the disordered state of the injured site. In this article, we review the pathogenesis of oxidative stress in vitiligo and promising strategies for its treatment.

## 1 Introduction

Vitiligo is a refractory and disfiguring skin condition with a complex and diverse pathogenesis that includes genetic, environmental, oxidative stress, and autoimmune factors. Studies have shown that oxidative stress in the skin can lead to melanocyte damage and vitiligo onset and progression (Khan et al., 2009; Glassman, 2011). Patients with vitiligo have intrinsic melanocyte defects, as evidenced by the enlarged endoplasmic reticuli, mitochondrial structural abnormalities, and lower-than-normal peroxidase levels in them. Oxidative stress in organs such as the brain, heart, kidneys, liver, spine, and testes is often accompanied by cellular damage, inflammation, and metabolic derangements (Stavely and Nurgali, 2020).

Traditional treatments for vitiligo include topical or oral glucocorticoid therapy, ultraviolet irradiation phototherapy, and topical calcium-modulated phosphatase inhibitors (Rashighi and Harris, 2017; Qi et al., 2021; Okoro et al., 2023). Glucocorticoids (GC) can inhibit the activity of phospholipase A2, thereby blocking the conversion of membrane phospholipids to arachidonic acid and reducing the production of the inflammatory protein prostaglandin; in addition, GC can traverse the cell membrane to bind to cytoplasmic glucocorticoid-receptor complexes that inhibit transcription of inflammation-related cytokine transcription factors after binding to specific DNA binding sites. Thus, glucocorticoid-receptor complexes binds directly and inhibits the AP-1 transcription factor and inhibits the NF-κB in a yet unclear manner. However, the success rate of glucocorticoid therapies varies greatly and they often cause acne, skin atrophy, capillary dilation, and complications such as hirsutism (Ahluwalia, 1998; Radakovic-Fijan et al., 2001; Gabros et al., 2023). UV light therapy induces differentiation of CD4^+^CD25^−^T cells into regulatory T cells by stimulating epidermal expression of IL-10, which inhibits the activity of self-reactive T cells that destroy melanocytes, and also promotes melanocyte development, proliferation, and migration (Chen et al., 2003). However, this therapy has a long course and poses an increased risk of skin malignancies and adverse effects such as pruritus, erythema, and dryness. In addition, patients undergoing the treatment need to limit sun exposure to avoid inadvertently increasing the body’s cumulative dose of UV light (Esmat et al., 2017; Doghaim et al., 2019; Zubair and Hamzavi, 2020). The Vitiligo Working Group recommends an initial dose of 200 mJ/cm^2^ and can be treated two or three times a week (Mohammad et al., 2017). Topical calcium phosphatase inhibitors (TCL) such as tacrolimus 0.1% ointment and pimecrolimus 1% cream inhibit dephosphorylation of nuclear factors in activated T cells and inhibit the production of pro-inflammatory cytokines such as TNF-α, IL-2, IL-3, and IL-5, ultimately suppressing autoimmunity. However, patients using TCLs frequently experience relapses accompanied by local skin burning sensation, pruritus, acne, and other adverse effects (Seirafi et al., 2007; Sisti et al., 2016; Seneschal et al., 2021). Moreover, the above treatments need to be used in combination regimens, complicating vitiligo treatments. Therefore, finding a new efficacious treatment with few side effects is essential.

MSCs, as ideal seed cells for organ repair in regenerative medicine, have the ability to ameliorate oxidative stress and induce immunosuppression and repair of the microenvironment through cell-cell interactions and paracrine effects (Kusuma et al., 2022; Zhao et al., 2022). The International Society for Cellular Therapy sets a minimum standard of MSCs as follows: cells that are positive for the surface markers of CD73, CD90, CD29, and CD105, but not with CD14, CD45, CD34, CD11b, or human leukocyte antigen-DR; are plastic-adherent and fibroblast-like under standard culture conditions; and can differentiate into chondrocytes, adipocytes and osteoblasts *in vitro* (Ong et al., 2021). As a means of regenerative medicine to treat clinical diseases, MSCs repair and improve the microenvironment of lesions by secreting a variety of cytokines. Besides, due to the low expression of costimulatory molecules and class II major histocompatibility complex of MSCs, transplantation of MSCs does not trigger a strong immune rejection response. MSCs can self-renew and possess multi-directional differentiation abilities; they can be isolated from a variety of tissues and are suitable for autologous transplantation. Studies have shown that MSCs can accumulate at tissue damage sites through a homing effect to participate in repair processes (Gholamrezanezhad et al., 2011). Oxidative stress is widely present in Alzheimer’s disease (AD), and transplantation of human placenta amniotic membrane-derived mesenchymal stem cells (hAMMSCs) into C57BL/6J-APP transgenic mice can reduce lipid peroxidation and oxidative stress levels and increase antioxidant enzyme levels to ameliorate the oxidative stress damage characteristic of AD (Jiao et al., 2016). In a study, doxorubicin-induced SD rats injected with human bone marrow mesenchymal stem cells (BMMSCs) via the tail vein showed that BMMSCs were able to reduce oxidative stress, levels of ROS, and lipid oxidation in the rat kidneys (Song et al., 2018). Similarly, treatment with BMMSCs from C57 mice suppressed the oxidative stress in the nervous system of a C57 mouse model of human multiple sclerosis (Lanza et al., 2009). Additionally, C57 mouse adipose tissue-derived MSCs improved oxidative stress in a C57 mouse model of vitiligo (Bian et al., 2022). An effective treatment for vitiligo needs to provide three main effects: autoimmune response suppression, oxidative stress reduction, and melanocyte production stimulation. Stem cell therapy seems to provide all three of these factors simultaneously. Moreover, MSCs combined with narrow-spectrum medium-wave UVB (NB-UVB) therapy or co-cultured with autologous melanocytes have shown good therapeutic effects for vitiligo treatment in mouse models (Lim et al., 2014; Bian et al., 2022). Few studies on the treatment of vitiligo with MSCs exist, the therapeutic mechanisms have not been fully elucidated, and clinical data is lacking; however, the unique regenerative and repairing ability of MSCs and their paracrine regulatory effects are promising characteristics for the treatment of vitiligo.

## 2 ROS and oxidative stress in melanocytes

### 2.1 Melanogenesis accompanied by ROS production

**FIGURE 1:**
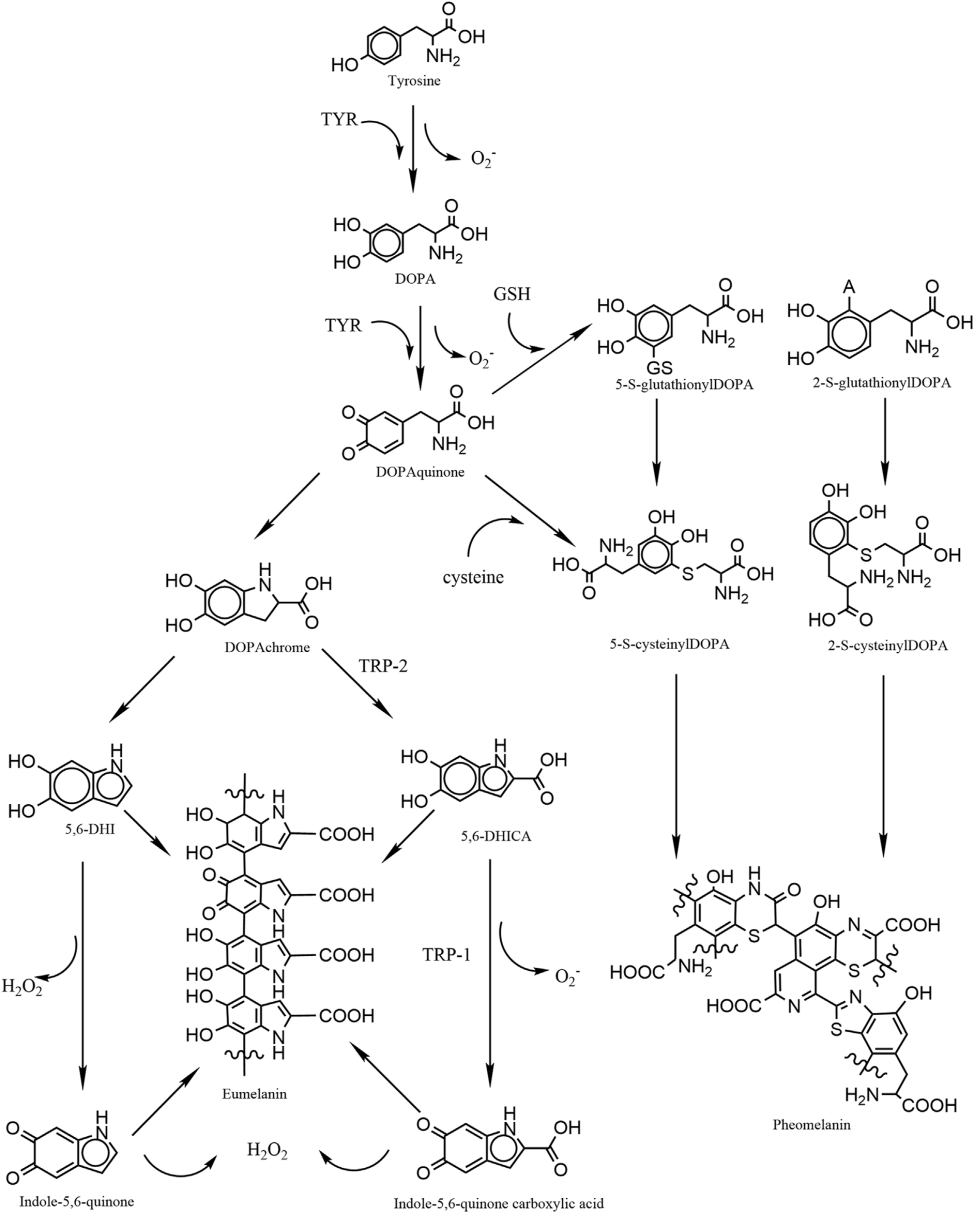
Graphic schematic showing the process of melanin synthesis in mammals, in which the two-step oxidation of tyrosine as well as the oxidation of DHI and DHICA are accompanied by the production of reactive oxygen species, which are potential contributors to oxidative stress in melanocytes.

Mammalian melanogenesis has two main pathways, one to produce eumelanin and the other pheomelanin. Phenylalanine is hydroxylated to L-tyrosine, which is itself hydroxylated to DOPA and further oxidized to dopaquinone (DQ). Hydroxylation of L-tyrosine is the rate-limiting step in melanin synthesis and is catalyzed by tyrosinase. At this point, if the amount of cysteine in melanocytes is high, DQ becomes pheomelanin, if the amount is low, DQ becomes eumelanin (Lin and Fisher, 2007; Ito and Wakamatsu, 2008). The higher the proportion of eumelanin, the darker the color of melanocytes. Therefore, Caucasians have a high proportion of pheomelanin, while black persons have the highest proportion of eumelanin. Melanosomes, which are organelles present in melanocytes, play a crucial role in the synthesis and storage of melanin. This pigment is subsequently transported from melanocytes to keratin-forming cells with the assistance of a melanin transporter protein. The primary function of melanin is to safeguard the skin against harmful UV radiation by absorbing it and preventing any potential damage. However, the process of melanogenesis leads to the generation of elevated levels of intracellular ROS as by-products. Notably, the oxidation of tyrosine to dopa and the subsequent oxidation of dopa to DQ both contribute to the production of superoxide anion (O_2_^.-^). During the conversion of DQ to eumelanin, DQ is first converted to dopa pigment via a redox exchange; next, through spontaneous decarboxylation reactions, the dopa pigment is either oxidized to indolequinone to produce 5,6-dihydroxyindole (5,6-DHI), or it is isomerized through the tyrosinase-related protein 2 (TRP-2) to produce 5,6-dihydroxyindole-2-carboxylic acid (5,6-DHICA), followed by the conversion of 5,6-DHICA to indole-5,6-quinone-2-carboxylic acid, and the conversion of 5,6-DHICA to indole-5,6-quinone in a redox reaction that generates hydrogen peroxide (Figure 1) (Agrup et al., 1982; Koga et al., 1992; Nappi and Vass, 1996; Ito and Wakamatsu, 2008; Rzepka et al., 2016). The ROS generated during melanin production renders melanocytes relatively susceptible to oxidative stress.

### 2.2 ROS trigger oxidative stress in melanocytes

ROS include O_2_^.-^, singlet oxygen (^1^O_2_), hydroxyl radicals (^·^OH), and perhydroxyl radicals (HO_2_^·^). During normal metabolic processes *in vivo*, ROS are produced by mitochondria and peroxisomes (Denat et al., 2014). Both intracellular and extracellular ROS production is associated with the activation of membrane-associated NADPH oxidase (NOX) and dual oxygenase (DUOX) (Diwanji and Bergmann, 2018). NOX and DUOX are proteins that transfer electrons across biological membranes; typically, they use NADPH as the electron donor and oxygen as the electron acceptor to undergo an electron transfer reaction and produce superoxide, which catalyzes the reduction of O^2^ to O^2-^ (Sies et al., 2017; Diwanji and Bergmann, 2018; Xia et al., 2021). The production of ROS is often triggered by pathological conditions such as inflammation and cancer, or by environmental stimuli such as ultraviolet radiation, cytotoxic chemicals, trauma, pregnancy, stress, and vaccinations (Boissy and Manga, 2004; Glassman, 2011; Denat et al., 2014; Picardo et al., 2015). Oxygen free radicals often cause molecular damage to macromolecules in the body and disrupt their biological functions, for example, ROS can denature proteins, disrupt lipids, cause aberrant cellular differentiations, activate apoptotic pathways, damage nuclear and mitochondrial DNA, and mediate the release of pro-inflammatory cytokines (Sander et al., 2004; Denat et al., 2014). Mitochondria is the most important endogenous source of ROS and the target of ROS-mediated damage. ROS are produced by mitochondria through the electron transport chain and are toxic by-products of oxidative phosphorylation. Under conditions of oxidative and antioxidant imbalance *in vivo*, ROS can lead to mitochondrial dysfunction, reduced energetic efficiency, and the production of excess ROS, which may lead to oxidative stress injury. Oxidative stress is characterized by an imbalance between the generation of free radicals and the body’s ability to counteract them, with a tendency towards oxidation. This imbalance can result in neutrophil inflammatory infiltration, elevated levels of protease secretion, and a significant production of oxidative intermediates. High concentrations of ROS trigger oxidative stress in the epidermis of patients with vitiligo. Studies have demonstrated that melanocytes in patients with vitiligo are more susceptible to oxidative stress than in individuals without vitiligo. The excessive ROS accumulation in the melanocytes of patients with vitiligo is due to both overproduction and insufficient clearance. ROS overproduction is mostly due to the environmental stimuli mentioned above. Insufficient clearance, by contrast, is associated with ROS removal mechanism abnormalities. Loss of catalase, glutathione peroxidase, and reduced intake of exogenous antioxidants like vitamin A and vitamin C contribute to the accumulation of ROS in cells (Schallreuter et al., 1991; Schallreuter et al., 1999).

### 2.3 Mitochondrial dysfunction/alteration of mitochondrial membrane lipids by ROS

Mitochondria not only generate ROS, but they are also susceptible to damage caused by oxidative stress. Mitochondria, the main sites of cellular aerobic respiration, control the energy supply of healthy cells and are required for cell survival. Damage to the mitochondria of melanocytes negatively affects their survival. ROS damages biological macromolecules such as lipids, leading to lipid peroxidation, a decrease in cardiolipin (CL), and an increase in cholesterol content (Dell'Anna et al., 2007; Dell'Anna et al., 2010; Chen et al., 2021). Detection of high malondialdehyde (MDA) levels is an indicator of lipid peroxidation in oxidative stress in the vitiligo skin lesions of patients. CL is a dimeric structure consisting of four fatty acyl chains present only in the mitochondrial membrane as its most abundant lipid (Dell'Anna et al., 2010). Studies have shown that cholesterol levels in the peripheral blood mononuclear cells of patients with vitiligo are significantly higher than in normal cells due to increased expression of the rate-limiting enzyme in cholesterol biosynthesis, 3-hydroxy-3-methylglutaryl coenzyme A reductase (HMG-CoA reductase) (Dell'Anna et al., 2010). The levels of CL in melanocytes and peripheral blood mononuclear cells in patients with vitiligo are significantly lower than those in normal cells. CL is the main membrane lipid of the mitochondrial electron transport chain (ETC.) complex, and a decrease in CL leads to an impairment in the activity of the mitochondrial, ETC., complex I (CXI), which increases ROS accumulation, and leads to the peroxidation of the mitochondrial membrane, further exacerbating CL oxidation, leading to a reduction in its content, and ultimately causing the death of melanocytes (Dell'Anna et al., 2007; Dell'Anna et al., 2010). Some genes within mitochondria, such as SIRT3 and TROM2, have been associated with oxidative stress. ROS activation of SIRT3 within mitochondria leads to mitochondrial fragmentation, and activation of TROM2 leads to mitochondrial calcium in-flow, both processes impairing the respiratory chain function within mitochondria, further contributing to the apoptosis of melanocytes in patients with vitiligo. Damage to the respiratory chain results in mitochondria producing high ROS levels (Dell'Anna et al., 2017). The altered lipid of the mitochondrial membrane leading to impaired mitochondrial activity may be the reason for the increased susceptibility of mitochondria to ROS (Dell'Anna et al., 2010).

## 3 Mechanism of antioxidant action of MSCs and research progress in vitiligo

### 3.1 Research on MSCs therapy for vitiligo

MSCs are a type of non-hematopoietic adult stem cell, derived from the mesoderm. They are primarily found in connective tissues and are abundant in adult bone marrow and adipose tissue. Additionally, they can be located in neonatal umbilical cords and placentas. At present, MSCs can be extracted from various sources including bone marrow, fat, amniotic fluid, membranes, umbilical cords, placenta, and peripheral blood. These cells possess diverse therapeutic functions and demonstrate anti-inflammatory and anti-apoptotic activities (Ramasamy et al., 2007; Uccelli et al., 2008; Hass et al., 2011; Elahi et al., 2016; Naji et al., 2019). While stem cells were initially utilized for cell therapy with the objective of differentiating and repairing damaged cells or tissues, the paracrine effects of mesenchymal stem cells are now more commonly employed to treat a diverse range of diseases. MSCs secrete a variety of cytokines involved in immunomodulation, such as TGFβ1, HGF, IL-10, PGE2, HO-1, and IL-6 (Uccelli et al., 2008; Kolios and Moodley, 2013; Jin, 2017). Moreover, cytokines secreted by MSCs can regulate the tis-sue repair microenvironment and promote regenerative repair through angiogenesis and apoptosis prevention (Uccelli et al., 2007; Boor and Floege, 2011). C57 mouse adipose tissue-derived MSCs combined with NB-UVB therapy or co-transplanted with autologous melanocytes in a mouse vitiligo model showed significant reductions in the white patch areas, the incurable rates, and the pigmentation score of vitiligo mice (Lim et al., 2014; Bian et al., 2022). However, the therapies are still in the preclinical animal experimental exploration stage, and we found no published clinical studies or reports. In the next paragraphs we will describe the potential application of the MSC paracrine function in vitiligo treatments.

### 3.2 Expression of HO-1 by MSCs exerts antioxidant effects in the Nrf2 signaling pathway

**FIGURE 2:**
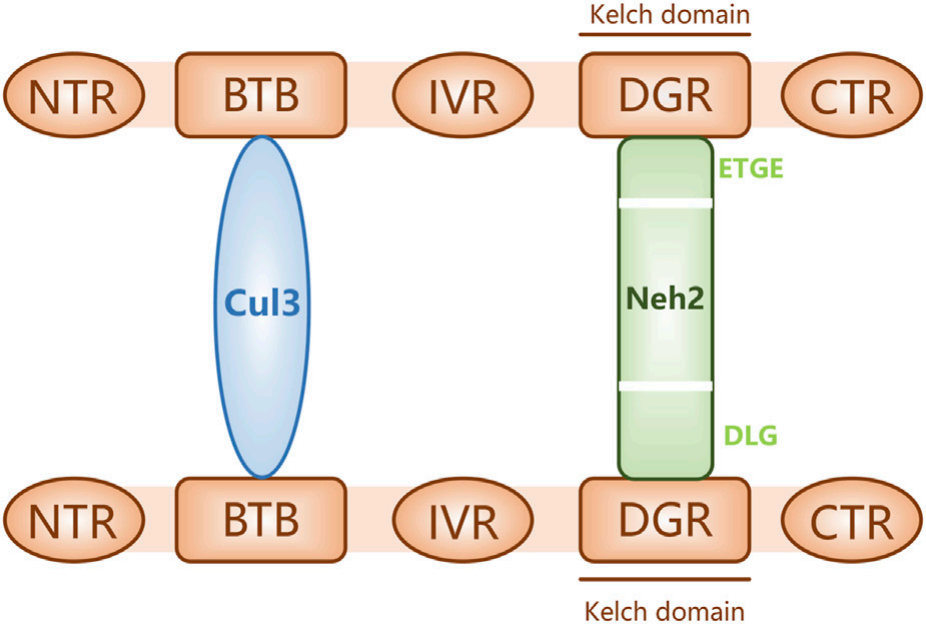
Keap1 bound to proteins Nrf2 (through its Neh2 domain) and Cul3. The schematic shows three structural domains (N-terminal region [NTR], intervening region [IVR], and C-terminal region [CTR]) and two feature domains (BTB and DGR) of Keap1.

**FIGURE 3:**
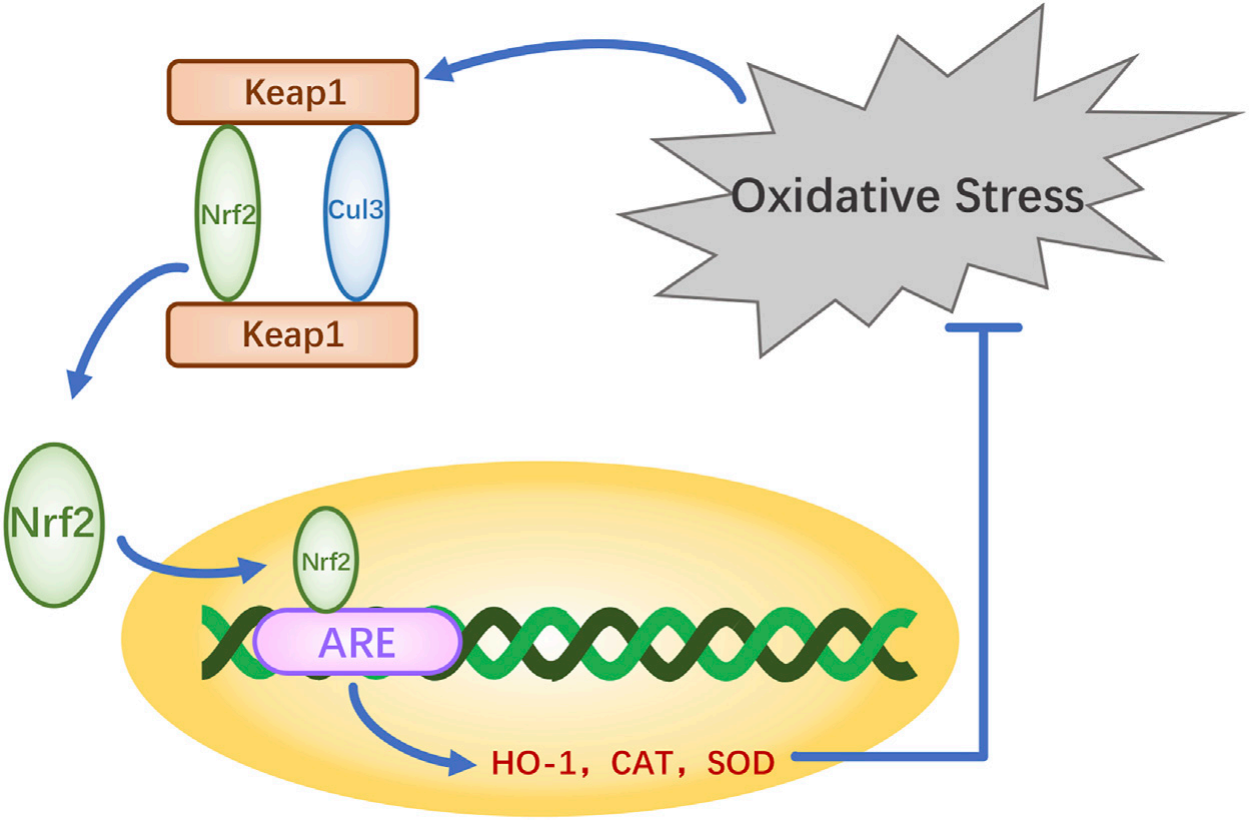
Molecular regulatory pathways underlying the antioxidant mechanisms of Nrf2.

The nuclear factor erythroid-2-related factor 2 (Nrf2)-Kelch-like ECH-associated protein 1 (Keap1)-antioxidant response element (ARE) pathway is one of the key antioxidant systems of MSCs. Signaling in the Nrf2-Keap-ARE pathway has been shown to be impaired in human vitiligo melanocytes, and re-establishing normal signaling may be a vitiligo treatment strategy (Jian et al., 2014; Lin et al., 2020). Nrf2 is a modular protein of 605 amino acids with seven structural domains (Neh1 to Neh7). Neh1 has a DNA-binding motif. Neh3, Neh4, and Neh5 bind coactivators and are trans-activating structural domains of Nrf2. Neh2, Neh6, and Neh7 all regulate Nrf2 stability; Neh2 has binding sites for two Keap1 cytoplasmic proteins (ETGE and DLG) and it negatively regulates Nrf2 transcriptional activity (Tong et al., 2006; Canning et al., 2015). Keap1 contains five regions, two of which are characterized domains, the broad-complex tramtrack and bric-a-brac (BTB) dimerization structural domain and the double glycine repeat (DGR) structural domain, also called the Kelch region. In addition, there are three other structural domains on Keap1: the N-terminal region (NTR), the intervening region (IVR), and the C-terminal region (CTR) (Figure 2). Keap1 binds the Neh2 domain of Nrf2 through the DGR domain. The inactivated Keap1 is usually bound to Nrf2 in the cytoplasm, where it gets ubiquitinated and then degraded. Under oxidative stress, Nrf2 uncouples from Keap1 and enters into the nucleus where it binds ARE, this complex activates the transcription of downstream genes to express related proteins such as heme oxygenase-1 (HO-1), catalase (CAT), and superoxide dismutase (SOD), which have antioxidant roles and protect cells from oxidative stress damage (Figure Figure3) (Schäfer et al., 2012; Canning et al., 2015; Telorack et al., 2016). During Nrf2 ubiquitination, the BTB region of Keap1 binds to the ubiquitin ligase Cullin3 on the E3 ubiquitin ligase complex, and the DGR region binds to Nrf2, ligating Nrf2 to the E3 ubiquitin ligase complex and transferring ubiquitin from the E3 ubiquitin ligase complex to the lysine residues between the ETGE and DLG motifs in Nrf2. The ubiquitinated Nrf2 is delivered to the proteasome for rapid degradation (Cloer et al., 2018). HO-1 protects human melanocytes against H_2_O_2_-induced oxidative stress damage through the Nrf2/ARE signaling pathway. In addition, experiments co-culturing human melanocytes treated with hydrogen peroxide and MSCs showed that MSCs promote melanocyte proliferation and upregulate the expression of the Nrf2 gene in both normal and oxidative-stressed melanocytes, suggesting that MSCs can significantly ameliorate oxidative stress injuries (Zhu et al., 2020). Moreover, MSC-derived exosomes can repair skin damage caused by oxidative stress through adaptive modulation of the Nrf2 defense system, mainly by inhibiting Nrf2 signaling as suggested by results in animal models (Wang et al., 2020; Zhu et al., 2020).

### 3.3 MSCs increase AKT phosphorylation in the PTEN signaling pathway

**FIGURE 4:**
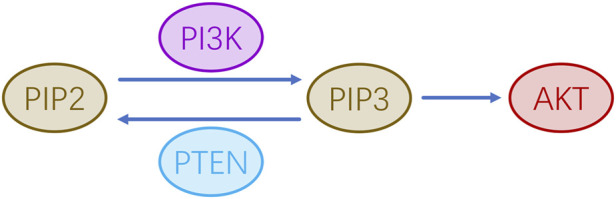
AKT activation is regulated by PI3K and PTEN.

PTEN is a tumor suppressor gene to mutations or loss leading to loss of function that can lead to tumors detected in patients with advanced cancers. The protein kinase B (AKT) gene is located downstream of the PI3K pathway genes, it encodes a serine/threonine proto-oncogene kinase involved in cell growth and survival. Phosphatidylinositol 3-kinase (PI3K) can phosphorylate the metabolite PIP2 to PIP3, which acts as a second messenger to recruit activated AKT, thus further activating the downstream regulatory pathway. By contrast, PTEN dephosphorylates PIP3 back to PIP2, thus terminating the PI3K signaling pathway, and indirectly inhibiting the AKT activities and signaling (Figure 4). PTEN mutations or deletions are observed in approximately 30% of human melanomas (Conde-Perez and Larue, 2012). High PTEN expression leads to reduced PI3K-mediated phosphorylation of AKT, melanocyte dysfunction or death, and may ultimately lead to depigmentation and vitiligo (Esquivel et al., 2020; Zhu et al., 2020). An immunohistochemical analysis by Zhu et al. showed that the PTEN expression in the skin of patients with vitiligo was significantly higher than that in normal skin. Melanocytes extracted from human foreskin tissue (passed on to the second or third generation) transfected with a PTEN expression plasmid and co-cultured with human BMMSCs resulted in the BMMSCs down-regulating their PTEN expression (Zhu et al., 2020). In addition, BMMSCs can reduce PTEN expression in the presence of oxidative stress *in vitro* in a 7,12-dimethylbenz [a]anthracene (DMBA)-induced leukemia cell model and a melanocytic vitiligo model, where they increased AKT phosphorylation and prevented oxidative stress-triggered cell death (Ahmed et al., 2019; Zhu et al., 2020). Phosphorylation inactivates PTEN (Vazquez et al., 2001; Das et al., 2003). IL-10 secreted by MSCs is involved in the PI3K/AKT pathway in melanocytes, it phosphorylates and inhibits PTEN function and induces AKT phosphorylation (Zhou et al., 2016). In addition, IL-10 treatment prevents the reduction of AKT phosphorylation that occurs after hydrogen peroxide (Zhou et al., 2016). In addition, MSCs promote melanocyte proliferation and reduce apoptosis of primary melanocytes in co-culture systems, and co-transplantation of MSCs with autologous melanocytes may be a promising therapeutic strategy for vitiligo (Zhu et al., 2020). Activation of the PI3K/AKT signaling pathway can also prompt Nrf2 to uncouple from Keap1 and translocate into the nucleus, activating downstream SOD, CAT, HO-1 and other antioxidant enzymes to indirectly protect melanocytes from oxidative stress (Huang et al., 2020; Yu and Xiao, 2021; Xuan et al., 2022). In summary, MSCs can regulate the proliferation of vitiligo melanocytes through downregulation of PTEN expression and activation of the PTEN/PI3K/AKT pathway, and they indirectly promote the Nrf2/HO-1 pathway to inhibit apoptosis of melanocytes and reverse the progression of vitiligo.

### 3.4 Antioxidant effects of IL-10 and HO-1 secreted by MSCs

**FIGURE 5:**
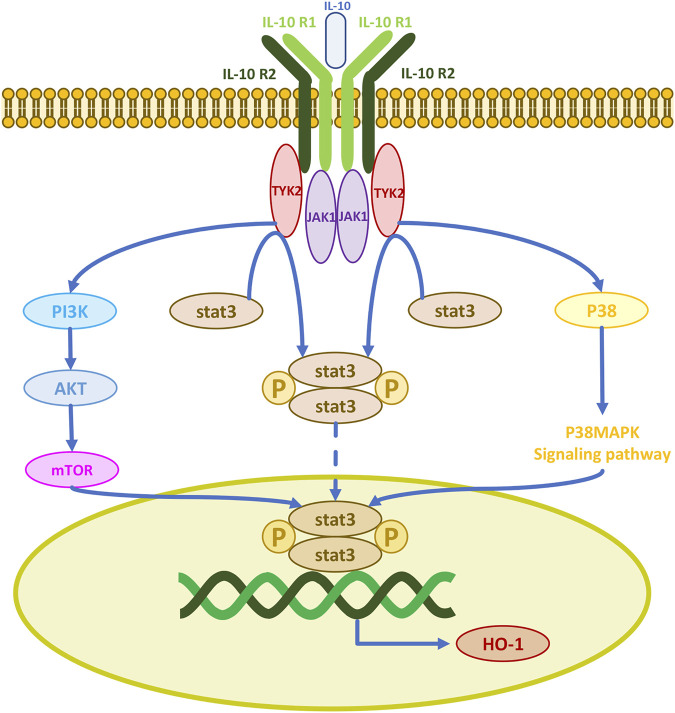
IL-10 activates three signaling pathways for cellular production of the antioxidant enzyme HO-1.

Cells rely on the availability of a range of enzymatic antioxidants (SOD, CAT, GPx) and small non-enzymatic antioxidants (GSH) to maintain the dynamic balance of redox and prevent the overproduction of free radicals. The therapeutic antioxidant roles of MSCs in models of erectile dysfunction, premature aging, pulmonary fibrosis, acute kidney injury, radiation aortic injury, and diabetes mellitus are mediated by the upregulation of antioxidant enzymes *in vivo* (Zarjou et al., 2011; El-Tantawy and Haleem, 2014; Ni et al., 2015; Ono et al., 2015; Xie et al., 2015; Shen et al., 2018; Yang et al., 2018). Thus, their antioxidant effects may be the result of free radical scavenging and upregulation of antioxidant enzymes, processes which enhance the antioxidant defenses of host tissues. IL-10 is a major regulator of the immune response with immunosuppressive and anti-inflammatory functions, it inhibits the production of several pro-inflammatory cytokines including tumor necrosis factor-alpha, interleukin-1, and interleukin-6 (Williams et al., 2004). IL-10 exerts its physiological effects mainly through the Janus kinase 1/signal transducer and activator of transcription 3 (JAK1/STAT3) pathway. The IL-10 receptor consists of two IL-10R1 and two IL-10R2 chains. When IL-10 binds to its receptor complex, the receptor is activated and transmits signals to the intracellular JAKs. There are four different JAKs in different receptor complexes, JAK1, JAK2, JAK3, and tyrosine kinase 2 (TYK2) (O'Shea and Plenge, 2012). The initial signal is transmitted to JAK1 and TYK2, as JAK1 is permanently associated with IL-10R1 and TYK2 with IL-10R2. The JAK kinase complex undergoes phosphorylation and induces phosphorylation of the downstream molecule STAT3, which can then enter the nucleus (O'Shea and Plenge, 2012; O'Shea et al., 2013). Thus, IL-10 induces HO-1 expression by activating STAT3 (Figure 5) (Ricchetti et al., 2004). IL-10 also upregulates AKT phosphorylation, which activates the transcription factor STAT3 (Dhingra et al., 2011). IL-10 activates PI3K and its downstream protein AKT initiating an activation cascade in sequence including mTOR, STAT3, and HO-1 production (Yokogami et al., 2000; Meng et al., 2019). HO-1 can also be produced after IL-10 induction of p38MAPK phosphorylation and STAT3 activation (Lee and Chau, 2002). HO-1 can be directly produced by MSCs, and most human MSCs express HO-1 (Chabannes et al., 2007). HO-1 is a heat shock stress-responsive protein induced by inflammatory stimuli and oxidative stress with anti-inflammatory and antioxidant functions. MSC-induced HO-1 protects cells from oxidative stress damage. The LPS-induced HO-1 is as effective and it inhibits lipopolysaccharide-induced proinflammatory cytokine expression and increases LPS-induced IL-10 expression in macrophages (Inoue et al., 2001; Chauveau et al., 2005; Chen et al., 2013). Since IFN-γ sends signals to JAK1 and JAK2, Stat1 is activated to produce CD8^+^ T cell chemokines CXCL9 and CXCL10. Therefore, JAK1/2 inhibitors such as ruxolitinib and baricitinib can currently be used to block the IFN-γ-CXCL9/10-CXCR3 axis to treat vitiligo (Bishnoi and Parsad, 2018; Feng and Lu, 2022; Diotallevi et al., 2023; Iwanowski et al., 2023).

Bcl-2 and Bax are proteins associated with apoptosis in the mitochondrial apoptotic pathway. Bcl-2 is an endogenous membrane protein with anti-apoptotic properties that inhibits apoptosis and cell death (Kowaltowski et al., 2004). Conversely, Bax is a pro-apoptotic molecule that triggers apoptosis by releasing cytochrome c prior to caspase activation and subsequent proteolysis. Following treatment with 100 μM of hydrogen peroxide for 3 h, the levels of Bax, cleaved-caspase 3, and cytochrome c increase in melanocytes, while the expression of Bcl-2 and Bclxl decreases (Zhou et al., 2016). In addition, pretreatment with IL-10 partially inhibits this pro-apoptotic effect of hydrogen peroxide (Zhou et al., 2016). These results suggest that the protective effect of IL-10 on melanocytes is partially mediated by promoting the expression of Bcl2 and Bclxl, decreasing the expression of Bax, restoring the balance between pro-apoptotic and anti-apoptotic peptides, and preventing the release of cytochrome c into the cytosol, thereby blocking the caspase-dependent apoptotic pathways (Zhou et al., 2016).

### 3.5 Mitochondrial transfer by MSCs to cells with defective mitochondria

Spees et al. first described mitochondrial donation from hMSCs, leading to the hypothesis that MSCs may transfer their mitochondria to mitochondria-damaged cells to improve their oxidative phosphorylation and alleviate ROS generation (Spees et al., 2006; Stavely and Nurgali, 2020). Studies using adipose tissue-derived MSCs co-cultured with ischemia-reperfusion-injured myocardial or vascular endothelial cells have revealed that mitochondria from damaged cells act as danger-signaling organelles that trigger the anti-apoptotic function of MSCs (Galluzzi et al., 2012; Mahrouf-Yorgov et al., 2017). Phagocytosis and degradation of damaged cell-derived mitochondria by MSCs induces production of HO-1 in MSCs to induce their production of new mitochondria (Piantadosi et al., 2008; Zheng et al., 2012; Figeac et al., 2014; Mahrouf-Yorgov et al., 2017). Thus, the donated mitochondria enhance the resistance to oxidative stress injury in damaged cells (Mahrouf-Yorgov et al., 2017). Islam et al. infused bone marrow MSCs into the airways of mice with acute lung injury and found that the MSCs transferred their mitochondria to the alveolar epithelial cells (Islam et al., 2012). Phinney et al. found that bone marrow MSCs also donate mitochondria to LPS-treated macrophages via secreted extracellular vesicles (Phinney et al., 2015). Lin et al. co-cultured human umbilical cord-derived MSCs with 143B osteosarcoma cells depleted of mitochondrial DNA (mtDNA) after long-term exposure to ethidium bromide and assessed the restoration of their mitochondrial function, demonstrating that the human umbilical cord-derived MSCs had transferred their own mitochondria to the mtDNA-depleted cells, forming a chimeric mitochondrial organelle in the recipient cells (Lin et al., 2015). Their study suggests that MSCs can be used as a potential therapeutic strategy for diseases associated with inherited mitochondrial defects to improve mitochondrial function by transfer of healthy mitochondria to cells with defects (Lin et al., 2015).

### 3.6 Some ancillary antioxidant effects regarding MSCs-based nanoparticles and MSC-derived exosomes

MSCs impregnated scaffolds/patches and MSC secreted exosomes may be a way to control oxidative stress. MSCs-based nanoparticles (NPs) have a wide range of biomedical applications in tissue regeneration. This method can reduce the loss of MSCs on the way to the injury site after MSCs are infusion, thus improving the efficiency of MSCs inoculated. In addition, NP also promotes the proliferation of MSCs and induces the differentiation of MSCs, such as electrospun collagen I scaffold or the combination of collagen and chitosan hydrogel can support MSC adhesion and proliferation (Dong et al., 2019; Nikolova and Chavali, 2019). AgNPs, AuNPs and reduced graphene oxide can all keep intracellular oxidative stress under control (Kim et al., 2011; Raghav et al., 2022). Most commonly used exosomes are isolated from Wharton`s jelly-derived MSCs, which have been shown to enhance activate AKT and Stat3 pathway (Raghav et al., 2022). Besides, MSC-derived exosomes downregulate Nrf2 after oxidative stress *in vitro* and *in vivo* (Wang et al., 2020).

## 4 Conclusion and future prospects

ROS in melanocytes have a role in the pathogenic mechanisms of vitiligo, and amelioration of the damage caused by oxidative stress to melanocytes is important for the treatment of vitiligo. Research on the use of traditional glucocorticoid therapy, phototherapy, and calcium-modulated phosphatase inhibitors is ongoing, but the limitations of these treatments are substantial. Studies have indicated the possible advantages of MSCs for the treatment of different diseases, and the development of regenerative medicine has brought new ideas for the treatment of vitiligo. MSCs are easily extractable MSCs with antioxidant properties, and we expect to see treatments for vitiligo and other oxidative stress-related diseases based on the application of these cells, which promise to be highly effective with mild if any side-effects. In this paper, we reviewed the possible mechanisms of MSCs against oxidative stress on the basis of diverse signaling pathways and the evidence from studies on the novel mitochondrial donation of MSCs. The strategies for MSC treatment of vitiligo are still being explored *in vitro* and in animal experiments, and clinical reports are not available yet to comment on the feasibility and safety of MSC treatments. Concerns on possible tumorigenicity and immune rejection, as well as the needed dosage, route, and frequency of administration of MSC therapy remain to be investigated before the clinical value of MSCs can be assessed.
